# Construction of Pseudomolecules for the Chinese Chestnut (*Castanea mollissima*) Genome

**DOI:** 10.1534/g3.120.401532

**Published:** 2020-08-26

**Authors:** Jinping Wang, Shoule Tian, Xiaoli Sun, Xinchao Cheng, Naibin Duan, Jihan Tao, Guangning Shen

**Affiliations:** * Shandong Institute of Pomology, 66 Longtan Road, Tai’an Shandong, 271000, China; † Biomarker Technologies Corporation, Floor 8, Shunjie Building, 12 Fuqian Road, Nanfaxin Town, Shunyi District, Beijing, 101300, China; ‡ Shandong Centre of Crop Germplasm Resources, Shandong Academy of Agricultural Sciences, Jinan, Shandong, 250100, China

**Keywords:** Chinese chestnut, *Castanea mollissima* Blume, genome assembly, single molecular sequencing, high-throughput chromosome conformation capture

## Abstract

The Chinese chestnut (*Castanea mollissima* Bl.) is a woody nut crop with a high ecological value. Although many cultivars have been selected from natural seedlings, elite lines with comprehensive agronomic traits and characters remain rare. To explore genetic resources with aid of whole genome sequence will play important roles in modern breeding programs for chestnut. In this study, we generated a high-quality *C. mollissima* genome assembly by combining 90× Pacific Biosciences long read and 170× high-throughput chromosome conformation capture data. The assembly was 688.93 Mb in total, with a contig N50 of 2.83 Mb. Most of the assembled sequences (99.75%) were anchored onto 12 chromosomes, and 97.07% of the assemblies were accurately anchored and oriented. A total of 33,638 protein-coding genes were predicted in the *C. mollissima* genome. Comparative genomic and transcriptomic analyses provided insights into the genes expressed in specific tissues, as well as those associated with burr development in the Chinese chestnut. This highly contiguous assembly of the *C. mollissima* genome provides a valuable resource for studies aiming at identifying and characterizing agronomical-important traits, and will aid the design of breeding strategies to develop more focused, faster, and predictable improvement programs.

The Chinese chestnut (*Castanea mollissima* Blume), of the Fagaceae family (Figure 1), is one of the most anciently (>3500 years) domesticated and a multipurpose tree species (Zhang *et al.* 2005). It is cultivated in East Asia, primarily in China, Korea, and Vietnam (Huang *et al.* 1999). *C. mollissima* is a dominant ecological and economic tree (Zhang *et al.* 2005) which typically grows and flourishes in mountainous regions with poor soil. This species exhibits resistance to two major pathogens, *Cryphonectria parasitica* and *Phytophthora cinnamomi*, both responsible for the complete demise of the susceptible American chestnut (*Castanea dentata*), a dominant US forest species (Anagnostakis 2012; Fang *et al.* 2013). Thus, *C. mollissima* could be a useful genetic donor for the improvement of the resistance of American chestnuts to these pathogens (Fang *et al.* 2013). In addition to its ecological value, the nuts produced by *C. mollissima* are very delicious and contain many nutrients, including starch, protein, fat, fiber, calcium, phosphorus, and iron. Due to the high starch content and high nutritional value of the nuts, *C. mollissima* is categorized as a “woody grain and oil” plant (Li *et al.* 2016; Zhu 2016; Hong and Feng 2017). Over 300 *C. mollissima* cultivars are currently used for nut production. These cultivars are subdivided into five major regional groups: Northern, Yangtze River Valley, Sichuan and Guizhou, Southern, and Southwestern (Zhang *et al.* 2005). More than two million tons of *C. mollissima* nuts are produced in China each year (China-statistical-yearbook, http://www.shujuku.org/). However, as most of the cultivars have been artificially selected directly from natural chestnut seedlings, elite lines with comprehensively selected character sets (*e.g.*, nut quality, production, and processing adaptabilities) are still needed.

**Figure 1:**
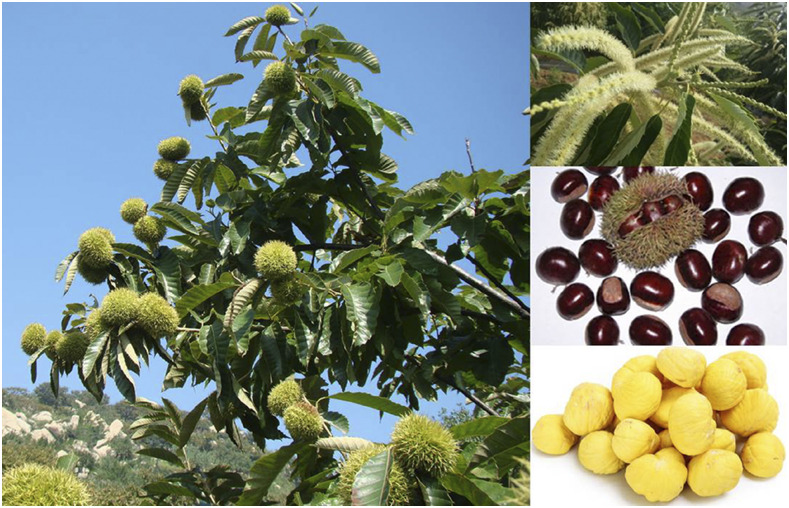
Tree, flowers and nuts of *Castanea mollissima*.

Because of the above important values, high-quality genomes are needed. The previous genome, especially the one supported by Staton *et al.* (2015) has played a huge role in the early stage of Chinese chestnut researches and utilization, such as in disease resistance QTL (Quantitative Trait Loci) location (Kubisiak *et al.* 2013). However, the existing genomic data of *C. mollissima* (Staton *et al.* 2015, 2019; Xing *et al.* 2019) are still fragmented and with no complete chromosomal information, due to the high heterozygosity of the genome and technical limitations. In order to identify important structural and functional elements of genomes and use valuable genetic variation for breeding, accurate, complete and contiguous genome assemblies are essential (Love *et al.* 2014). Previous studies have shown that combining Pacific Biosciences (PacBio) and Illumina data significantly improve N50 length and generate more accurate assemblies (Koren *et al.* 2012; Jiao *et al.* 2017; Westbrook *et al.* 2019). High-throughput chromosome conformation capture (Hi-C) is a cost-effective technique for assigning, ordering, and orienting genomic sequences onto chromosomes (Lin *et al.* 2018). This technique has been successfully used to improve the assemblies of several complex genomes, including animals (Bickhart *et al.* 2017), grain crops (Avni *et al.* 2017; Mascher *et al.* 2017), oil crops (Yin *et al.* 2018), and fruit crops (Tang *et al.* 2019). We therefore constructed a contiguous genome assembly of *C. mollissima*, using a combination of PacBio long reads, and Hi-C data which provides a valuable source of genomic data for future molecular and breeding researches of the Chinese chestnut.

The burrs of chestnut fruits have very sharp spines (Zhang *et al.* 2005), which often harm chestnut farmers. Indeed, there are many reports of chestnut fruits stuck on farmers’ eyes (Zhang *et al.* 2013; García-García *et al.* 2016). Also, the varieties with thin burr are popular in chestnut production areas for their high productivity. Although Zhang once reported natural varieties with no spines or degenerated spines on burr (Zhang *et al.* 2005), we have been unable to locate these natural varieties in the described region. Thus, we hope that based on the assembly, genes controlling burr development could be identified in order to promote burr trait improvement.

## Materials and Methods

### Sample collection, library construction and sequencing

In order to get the common genome characteristic of *C. mollissima*, and eliminate interference from rootstocks with different genotypes, a seedling cultivar line N11-1 was selected as the sequencing sample. This line exhibits not only the common phenotype of *C. mollissima*, but also excellent characteristics such as high yield and high sugar content. Thus, it was selected as a potential variety, and further studies will be carried out on its excellent traits and pedigree. It is now conserved at the Wanjishan Experimental and Demonstration Station of Shandong Institute of Pomology, Tai’an, Shandong, China (36.206 E, 117.084 N). Genomic DNA was extracted from the leaves using the cetyltrimethyl ammonium bromide (CTAB) method, as described in the PacBio shared protocol (Rhoads and Au 2015). The quality of the extracted genomic DNA was assessed using 1% agarose gel electrophoresis.

All sequencing was performed by Biomarker Technologies Corporation (Beijing, China). For Illumina sequencing, paired-end (PE) library with insert sizes of ∼350 bp was constructed following the protocol provided by Illumina Nova seq 6000 (San Diego, CA, USA). In total, 54.55 Gb of cleaned PE sequences were generated (Supplementary Table S1). The cleaned sequences were used for genome feature characterization (including genome size and heterozygosity level), assembly correction, and assembly evaluation.

Long read sequencing was performed on a PacBio Sequel sequencer (Pacific Biosciences, Menlo Park, CA, USA) following the manufacturer’s instructions (Rao *et al.* 2014). Genomic DNA were mechanically sheared and polished with DNA-damage repair and end-repair enzymes, followed by blunt-end ligation and exonuclease treatment. Fragments >10 kb were selected using Blue Pippin (Sage Science, Inc., Beverly, MA, USA), and used to generate the sequence library. A total of six SMRT (Single Molecule Real-Time) cells and 66.98 Gb of filtered subreads longer than 12 kb were used for assembly (Supplementary Table S2, Supplementary Figure S1).

We constructed Hi-C fragment libraries with insert sizes of 300-700 bp as illustrated in Rao *et al.* (2014). In brief, nuclear DNA was cross-linked *in situ*, extracted, and then digested with DpnII. The sticky ends of the digested fragments were biotinylated, diluted, and then randomly ligated to one another. Biotinylated DNA fragments were enriched, re-sheared, and then used to prepare the sequencing library, which was sequenced on Illumina Nova seq 6000 (San Diego, CA, USA).

RNA for transcriptome sequencing was extracted from *C. mollissima* tissues including leaves, stems, flowers (inflorescences), fruits, and roots, using a PureLink RNA Mini Kit (Thermo Fisher Scientific, Carlsbad, CA, USA) following the manufacturer’s instructions. Pools of total RNA from all sampled tissues were used to prepare RNA-Seq libraries using TruSeq RNA Sample Preparation Kits (Illumina, San Diego, CA, USA) following the manufacturer’s instructions. The prepared libraries were sequenced on an Illumina HiSeq 2500 platform (PE, 100-bp reads), generating approximately 10.3 Gb of data. These RNA-Seq data were used for genome annotation and to assess the completeness of the genome assembly.

### Genome assembly

*C. mollissima* genome size (G) was initially estimated based on k-mer frequency (Kim *et al.* 2011), using the formula G = (T*k* - A*k)/*D*k*, where T*k* was the total number of *k*-mers, A*k* was the number of abnormal *k*-mers (those either too frequent or too infrequent), and D*k* was the average *k*-mer depth. The sequencing depth was estimated by determining the highest peak value of the frequency curve of the K-mer occurrence distribution.

The PacBio reads were first corrected using the error correction module of Canu v1.5 (https://github.com/marbl/canu). In this correction step, Canu was used to select longer seed reads with the settings “genomeSize = 700000000” and “corOutCoverage = 60,” and overlapping raw reads were detected using the highly sensitive overlap identifier MHAP (mhap v2.1.2; https://github.com/marbl/MHAP.git), with the option corMhapSensitivity set to “low/normal/high.” Error correction was then performed with Canu, using the falcon_sense method with correctedErrorRate set to 0.025. The error-corrected reads were trimmed by removing unsupported bases and hairpin adapters to obtain the longest supported range, using default parameters. The final cleaned reads were then assembled using Canu.

The PacBio raw reads were also *de novo* assembled using wtdbg v1.2.8 (https://github.com/ruanjue/wtdbg), with the command “wtdbg -i pbreads.fasta -t 64 -H -k 21 -S 1.02 -e 3 -o wtdbg.” We used the error-corrected reads from Canu to improve the assembly. Finally, a consensus assembly was generated using the command “wtdbg-cns -t 64 -i wtdbg.ctg.lay -o wtdbg.ctg.lay.fa -k 15.”

We merged the two assemblies generated by Canu and wtdbg using Quickmerge (Chakraborty *et al.* 2016, https://github.com/mahulchak/quickmerge.git), in order to integrate the advantages of the two different assemblers. In brief, we aligned the Canu-generated contigs to the wtdbg-generated contigs using MUMmer v4.0.0 (https://github.com/mummer4/mummer), with the nucmer parameter set “-b 500 -c 100 -l 200 -t 12” and the delta-filter parameter set “-i 90 -r -q.” Based on these alignments, the two set of contigs were merged using Quickmerge, with the parameter set “-hco 5.0 -c 1.5 -l 100000 −ml 5000.”

The merged assembly was polished with the Illumina reads using Pilon v1.22 (Pilon, RRID: SCR 014731), with the parameter set “-mindepth 10 -changes -threads 4 -fix bases.”

### Assembly revision and validation

We used Hi-C data to correct and optimize our initial assembly. The clean Hi-C reads were first truncated at the putative Hi-C junctions and then the resulting trimmed reads were aligned to the assembly results with BWA v0.7.10 (BWA, RRID: SCR 010910). Only uniquely alignable paired reads, with mapping quality >20, were retained for further analysis. Invalid read pairs, including dangling-end, self-cycle, re-ligation and dumped products, were filtered by HiC-Prov2.8.1 (https://github.com/nservant/HiC-Pro). The valid interaction pairs were used to correct the scaffolds. Scaffolds were clustered, ordered and oriented onto chromosomes by using LACHESIS (http://shendurelab.github.io/LACHESIS/).

Before chromosome assembly, we preassembled scaffolds to correct errors, which required splitting the scaffolds into segments average of 50 kb long. The Hi-C data were mapped to these segments using BWA. The uniquely mapped data were retained and assembled using LACHESIS (Burton *et al.* 2013). Placement and orientation errors exhibiting obvious discrete chromatin interaction patterns indicate misassemblies and were manually adjusted. We examined the interaction map for each contig and broke 173 that were possibly misassembled. The corrected scaffolds were assembled with LACHESIS using the following parameters: CLUSTER MIN RE SITES = 52, CLUSTER MAX LINK DENSITY = 2, CLUSTER NONINFORMATIVE RATIO = 2, ORDER MIN N RES IN TRUN = 46, and ORDER MIN N RES IN SHREDS = 42. The Hi-C heatmap of *C. mollissima* was generated using HiCplotter (Akdemir and Chin 2015), at a resolution of 100-kb.

We assessed the completeness of the *C. mollissima* assembly using the Benchmarking Universal Single-Copy Orthologs (BUSCO) (BUSCO, RRID: SCR 015008), against the embryophyta_odb 10 dataset. We also evaluated the completeness of the assembly by mapping Illumina PE short reads back to the assembled genome using BWA.

### Genome annotation

Protein-coding genes in the *C. mollissima* genome assembly were predicted using a combination of *ab initio*, homolog, and RNA-Seq gene prediction approaches. *Ab initio* predictions were performed using a combination of Genscan (Genscan, RRID: SCR 012902), Augustus v2.4 (Augustus, RRID: SCR 008417), GlimmerHMM v3.0.4 (GlimmerHMM, RRID: SCR 002654), GeneID (v1.4), and SNAP v2006-07-28 (SNAP, RRID: SCR 002127), using parameters trained against *Arabidopsis* gene models. Homolog-based gene prediction was performed with GeMoMa (v1.3.1; Keilwagen *et al.* 2016), using the protein databases of several plant species: *Arabidopsis thaliana* (GenBank accession no. GCA_000001735.2), *Oryza sativa* (GenBank accession no. GCA_001433935.1), *Fraxinus excelsior* (GenBank accession no. GCA_900149125.1), and *Quercus suber* (genome available from http://www.oakgenome.fr/; GenBank accession no. GCA_002906115.1). To predict protein-coding genes with RNA-Seq, we aligned the RNA-Seq data to the *C. mollissima* genome using Hisat v2.0.4, and assembled the genes into transcripts using Stringtie v1.2.3. TransDecoder v2.0, GeneMarkS-T v5.1 (RRID: SCR 011930), and PASA (PASA, RRID SCR 014656) were then used to identify protein-coding genes. Finally, we integrated the protein-coding genes predicted by each of the three methods (*ab initio*, homology, and RNA-Seq) using EVM v1.1.1 (RRID: SCR 014659).

The protein-coding genes were annotated against several databases, including GenBank non-redundant protein database (Nr), eukaryotic orthologous groups of proteins (KOG), the Kyoto Encyclopedia of Genes and Genomes (KEGG) (KEGG, RRID: SCR 001120), and TrEMBL, using the Basic Local Alignment Search Tool (BLAST v2.2.31; Altschul *et al.* 1990) with an e-value cutoff of 1E-5. Gene Ontology (GO) terms were assigned to the protein-coding genes using the BLAST2GO pipeline (Blast2GO, RRID:SCR 005828).

To predict pseudogenes, we used GenBlastA (https://github.com/pvanheus/genblastA_to_gff.git) to scan the *C. mollissima* genome for homologous sequences and to identify known protein-coding genes. Within those homologous sequences, we used GeneWise (GeneWise, RRID: SCR 015054) to search for frameshift mutations or premature stop codons. Gene sequences containing frameshift mutations or premature stop codons were considered pseudogenes.

A *de novo* repeat library was constructed based on the *C. mollissima* assembly using three software packages: LTR-FINDER (v1.0.5; https://github.com/xzhub/LTR_Finder.git), RepeatScout (v1.0.5; https://github.com/mmcco/RepeatScout.git), and PILER-DF v2.4 (Edger and Myers 2005). The predicted repeats were classified using PASTEClassifier v1.0 (Hoede *et al.* 2014) and merged with Repbase (Bao *et al.* 2015). Finally, using the resulting repeat database as the repeat library, we identified repetitive sequences in the *C. mollissima* genome using RepeatMasker v4.0.5 (RepeatMasker, RRID: SCR 012954) with the following parameters: “-nolow -no is -norna -engine wublast.”

### Comparative genomic analysis

We identified gene family clusters in the complete gene sets of C. mollissima and eight other plant species (*O. sativa*, *Solanum tuberosum*, *A. thaliana*, *Quercus robur*, *Casuarina glauca*, *Juglans regia*, *Populus trichocarpa*, and *Populus euphratica*) using OrthoMCL (v2.0.9; Li *et al.* 2003) Gene family clusters were identified by first eliminating all genes encoding proteins less than 50 aa long in the *C. mollissima* gene sets. Second, similar proteins were identified in the genomes of the nine plant species listed above. Third, identified genes were clustered using OrthoMCL to obtain single-copy gene families and species-specific gene families. A species-level maximum likelihood phylogeny was constructed based on the alignment of 863 concatenated single-copy family genes from the nine plant species using Phyml release 20151210 (Kimura 1981), with the bootstrap set to 1000. Simultaneously, the *C. mollissima* proteins identified in step one were searched against the *Q. robur* dataset using BlastP (with an evalue cutoff of 1E-10). After identifying the chromosome carrying of each gene, gene collinearity between the two genomes was determined using MCScanX (Wang *et al.* 2012) with the parameters “-s 10 -b 2.” The divergence time was estimated by MCMCtree in the PAML (4.7a package; Yang 1997) using the known divergence time of *C. glauca* and *J. regia*, and *O. sativa* and *A. thaliana* from the Time-Tree database. In addition, we inferred gene family expansion and contraction using CAFE V4.2 (Kim *et al.* 2011).

### RNA-Seq analysis of tissue-specific expression

We analyzed the RNA sequences of several tissues, including the root, stem, leaf, flower (inflorescence) and fruit. To identify genes associated with burr development, we analyzed both the entire fruit and fruit without the burr. RNA sequences were analyzed using BMKCloud (www.biocloud.net). RNA-seq short reads were aligned to the *C. mollissima* genome assembled in this study using HISAT2 v2.0.4 software, with one mapped location being selected randomly for reads mapped to multiple locations. Gene expression levels in terms of fragments per kilobase of transcript per million fragments mapped reads (FPKM) values (Florea *et al.* 2013), were computed with Stringtie v1.2.33. Genes were considered expressed if the FPKM was >0. Differentially expressed genes (DEGs) analysis were identified using DESeq2 (Love *et al.* 2014) with the FDR = 0.01 and fold change set to ≥ 2. GO annotations and KEGG pathway enrichment analyses were performed for the tissue-specific DEG using the Database for Annotation, Visualization and Integrated Discovery (DAVID), as implemented in BMKCloud. We also determined the GO annotations and KEGG pathway enrichments of genes expressed only in the whole fruit.

### Data availability

Raw data and assembled sequence data for *C. mollissima* are available via NCBI (Bioproject accession PRJNA559042). The assembly and annotation data of *C. mollissima* are in the Genome Warehouse in BIG Data Center under accession number GWHANWH00000000, which are accessible at https://bigd.big.ac.cn/gwh. Supplemental material available at figshare: https://doi.org/10.25387/g3.12858206.

## Results

### Genome size, heterozygosity and assembly

Based on the distribution of 19-mers among the Illumina HiSeq reads (*i.e.*, the 54.55 Gb cleaned reads from the ∼350 bp insert library). The genome (G) of *C. mollissima* was estimated to be 655.18 Mb, with approximately 0.78% heterozygosity. The k-mer distribution curve peaked at a depth of 70, with a k-mer number of 46,432,321,425 (Figure S2). The peak at a k-mer depth of 141 was a repetitive peak (*i.e.*, k-mers duplicated because of repetition). The data used for the 19-mer analysis represented approximately 83× coverage of the genome.

The 66.98 Gb PacBio long reads used for *de novo* assembly represented >80-fold coverage of the estimated *C. mollissima* genome. The average length of these PacBio reads was 13,408 bp; the longest read was 90,903 bp and the N50 was 22,633 bp (Supplementary Table S3). Using Canu v1.5, we generated a draft assembly of 994,461,872 bp (Supplementary Table S4); using wtdbg v1.2.8, the assembly was 782,890,668 bp. Subsequently, by Quickmerge, we got the initial assembly of 793.80 Mb, with 1,381 contigs and a contig N50 of 3.38 Mb (Supplementary Table S4), which was much larger than k-mer estimation (655.18 Mb).

### Genome assembly correction and validation based on chromatin interactions

Our Hi-C sequencing generated a total of 110.83 Gb cleaned data, representing ∼170-fold coverage of the *C. mollissima* genome, with 91.82% high-quality (Q30) bases (Supplementary Table S5). Approximately 89.63% of the Hi-C reads were mapped to the initial assembly, and 59.63% of the mapped read pairs were uniquely mapped (Supplementary Table S6). Of the 210.65 million unique paired alignments, 161.13 million (76.49%) were valid interaction pairs (Supplementary Table S7). Thus, the Hi-C sequences were sufficient for the refinement of the initial assembly.

**Figure 2:**
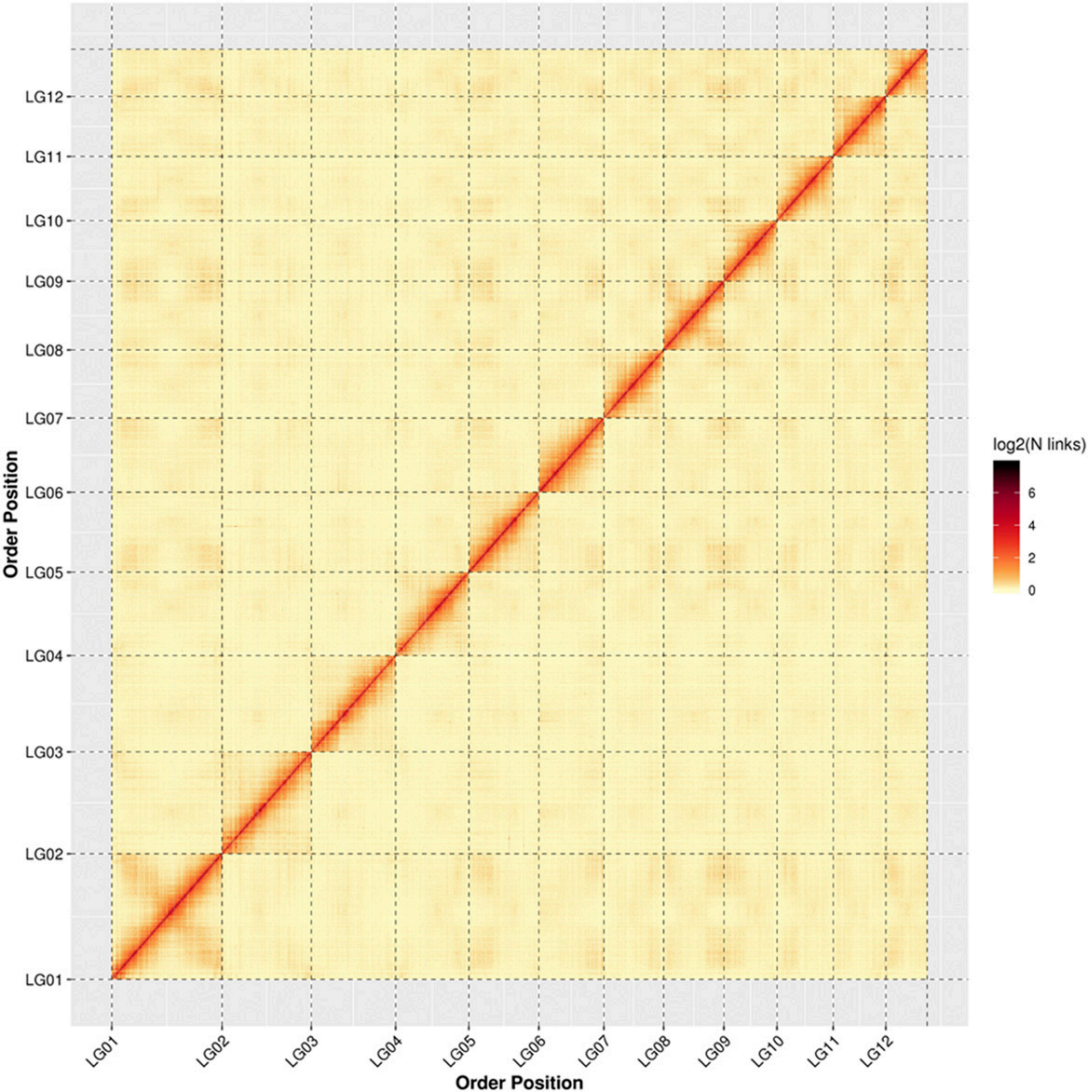
Distribution of the interaction frequencies among chromosomes. The distribution is based on the 170× high-throughput chromosome conformation capture (Hi-C) links.

After performing error correction using Hi-C sequence data, we generated a final assembly of the *C. mollissima* genome. The assembly was 688,929,566 bp long, with a contig N50 of 2.83 Mb (Table 1). In this assembly, 652 contigs (687,278,892 bp; 99.75%) were clustered onto 12 pseudochromosomes, corresponding to the 12 chestnut chromosomes. A total of 571 of the clustered contigs with a total length of 667,130,435 bp (97.07%) were correctly ordered and oriented (Table 2). The Hi-C heatmap of *C. mollissima*, generated by HiCplotter indicated that the frequency of intra-chromosome interactions decreased rapidly with linear distance (Figure 2). Compared with previously published genomes of *C. mollissima*, the additional mapping of the Hi-C data to the genome yielded a much more continuous, chromosome-level assembly (Table 3).

**Table 1:** Properties of the *Castanea mollissima* assembly

Contig number	Total contig length (bp)	Contig N50 (bp)	Contig N90 (bp)	Longest contig (bp)	GC content (%)
**671**	**688,929,566**	2,828,629	455,877	19,325,860	35.11

**Table 2:** Pseudochromosomes in the *Castanea mollissima* genome

Group	Number of contigs	Sequence length (bp)
Lachesis Group 1	52	90,647,674
Lachesis Group 2	84	74,427,361
Lachesis Group 3	97	70,533,118
Lachesis Group 4	72	62,883,648
Lachesis Group 5	60	60,035,541
Lachesis Group 6	38	53,752,763
Lachesis Group 7	55	51,743,333
Lachesis Group 8	35	50,879,406
Lachesis Group 9	41	45,391,892
Lachesis Group 10	51	46,531,048
Lachesis Group 11	38	44,129,845
Lachesis Group 12	29	36,323,263
Total Sequences Clustered	**652 (97.17%)**	**687,278,892 (99.75%)**
Total Sequences Ordered and Oriented	**571 (87.58%)**	**667,130,435 (97.07%)**

**Table 3:** Comparison of assembly quality in three genomes of *C. mollissima*

Parameter	*Castanea mollissima* genome assembly		
	I	II	III
Total sequence length (Mb) **Contigs**	**689.98**	**785.5**	**725.2**
Total contig No.	**671**	**2,707**	**15,892**
Contig N50 (kb)	2,828.6	944.5	96.7
Contig N90 (kb)	455.88	133.7	
Longest contig length (Mb)	19.3	6.58	
**Scaffolds**			
Total scaffold No.	**112**		**14,110**
Total scaffold length (Mb)	689.98		
Scaffold N50 (kb)	57,343.43		
Scaffold N90 (kb)	4,301.26		
Longest scaffold length (Mb)	90.2		
Anchored onto chromosome (Mb/%)	**687.3/99.75**		
Anchored with order and orientation (Mb/%)	**667.1/97.07**		

Footnotes: I: Present assembly; II: Assembly published by Xing *et al*; III: Assembly submitted by Clemson University Genomics Institute. The assembly II has no scaffold chromosome-level information, so the detailed comparisons were mainly on contig-level.

BUSCO analysis indicated that 92.44% of the core eukaryotic genes (1492 of 1614) were captured by the *C. mollissima* genome assembly, and that 88.79% (1,433 of 1614) were complete (Supplementary Table S8). We found that 99% of the Illumina PE short reads could be mapped, and more than 95% were properly mapped in pairs (Supplementary Table S9).

### Genome annotation

Using a combination of *ab initio*, homology, and RNA-Seq gene prediction approaches, we predicted 33,638 genes in the *C. mollissima* genome assembly. A total of 33,074 of the predicted genes were based on homology, and RNA-Seq predictions (Supplementary Tables S10, S11 and Figure S3), 97.73% were classified into Annotation database (Table S12). We identified 366,839,546 bp of repetitive sequences, accounting for 53.24% of the assembly, including 34.24% retrotransposons and 5.52% DNA transposons. Long terminal repeat (LTR) transposons (24.17%) were the largest family of repeats (Supplementary Table S13).

### Comparative genomic analysis of C. mollissima and other plant species

We determined gene family clusters in the complete gene sets of *C. mollissima* and eight other plant species (*O. sativa*, *S. tuberosum*, *A. thaliana*, *Q. robur*, *C. glauca*, *J. regia*, *P. trichocarpa*, and *P. euphratica*). Of the 33,638 genes in the *C. mollissima* genome, 30,192 were grouped into 15,367 gene clusters; 508 of these clusters were specific to *C. mollissima* (Table 4). The Chinese chestnut-specific gene families contained 3,201 genes. Of these, four genes (*EVM0002617*, *EVM0020045*, *EVM0029632* and *EVM0032868*) were associated with the plant-pathogen interaction pathway; three genes (*EVM0008748*, *EVM0019137* and *EVM0030327*) were associated with vitamin B6 metabolism; two genes (*EVM0007389* and *EVM0020714*) were associated with starch and sucrose metabolism; and one gene (*EVM0002118*) was associated with mismatch repair. An additional two genes (*EVM0004145* and *EVM0007164*) were functionally categorized in plant self-incompatibility protein S1 family.

**Table 4:** Gene families in the genomes of *Castanea mollissima* and eight other plant species

Species	Total genes	Genes clustered	Gene families	Unique gene families
*Casuarina glauca*	29,827	21,278	14,176	707
*Juglans regia*	36,263	31,861	14,833	738
*Solanum tuberosum*	39,027	31,534	13,407	1,421
*Oryza sativa*	38,852	25,505	12,626	2,016
*Populus euphratica*	30,612	28,991	16,042	88
*Arabidopsis thaliana*	27,369	23,120	12,960	743
*Quercus robur*	25,808	20,813	12,245	469
*Populus trichocarpa*	41,335	32,920	17,194	617
*C. mollissima*	**33,638**	30,192	15,367	508

**Figure 3:**
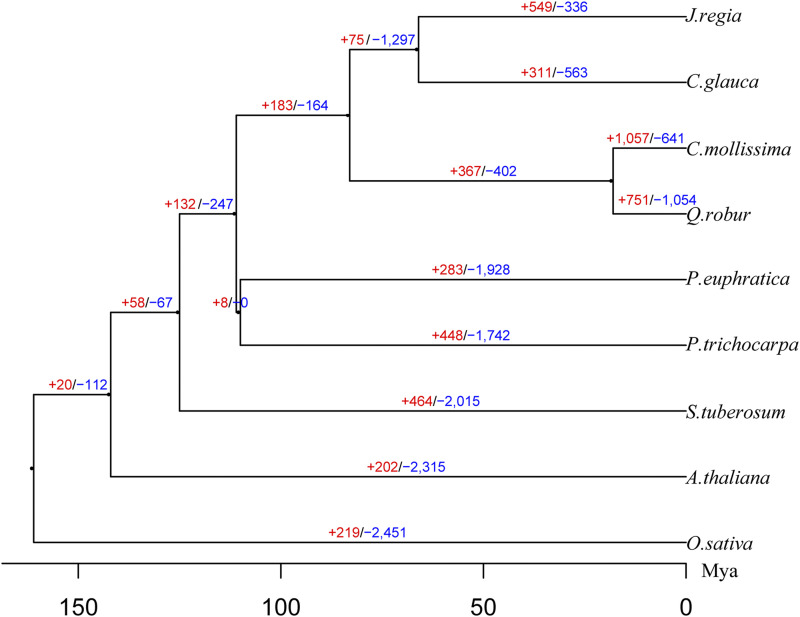
Species phylogenetic tree and gene family evolution. Numbers on the branch indicate counts of gene families that are under either expansion (red) or contraction (blue). The bootstrap is 1000. The bottom scale bar shows divergence time, Mya: million years ago.

The species-level maximum likelihood phylogeny, based on alignment of 863 concatenated single-copy family genes from nine plant species, recovered a sister relationship between *C. mollissima* and *Q. robur* (Figure 3). Next, the divergence time of *C. mollisima* and *Q. robur* was estimated to be ∼18.3 million years ago (Mya) (Figure 3). We identified 1,057 gene families that were expanded in the Chinese chestnut compared to other species (Figure 3).

**Figure 4:**
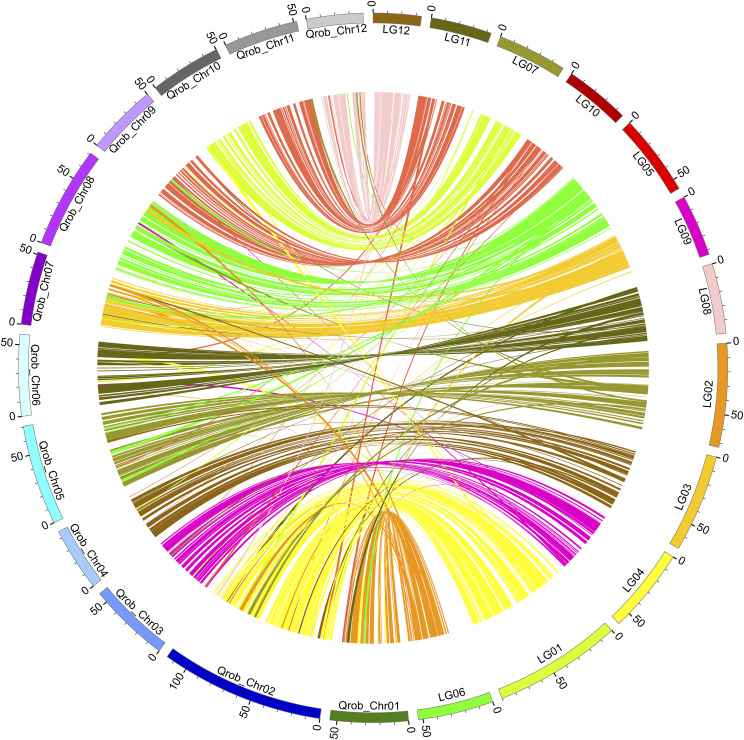
Genome synteny between the Chinese chestnut and the European oak. European oak chromosomes are labeled “Qrob_chr”; Chinese chestnut chromosomes are labeled “LG”.

As the assembly was finely anchored and oriented onto the 12 pseudochromosomes with the aid of Hi-C sequence data, MCScanX identified long syntenic blocks shared between the genomes of *C. mollissima* and *Q. robur* (Figure 4). There were 18,399 collinear genes, accounting for 30.95% of the total genes.

### Transcriptome analysis of tissue-specific expression

**Figure 5:**
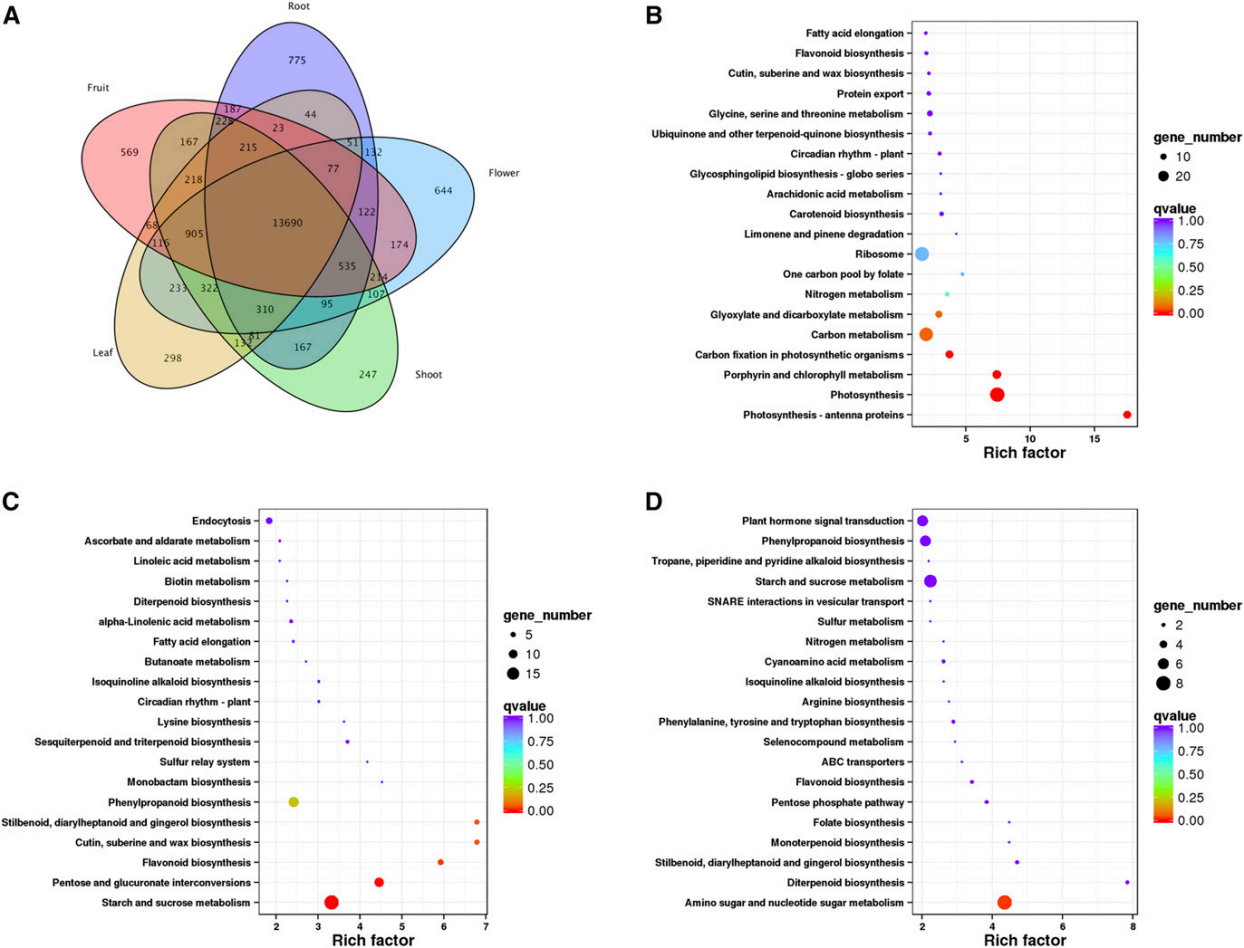
Tissue-specific gene analysis. (A) Venn diagram showing unique and shared genes among 5 tissues. Numbers represent the number of genes that were unique or shared. (B−D) KEGG enrichment of tissue-specific genes in leaf, flowers and fruit respectively. The node size represents the gene numbers enriched in each KEGG pathway. The color bar illuminates P-value from red (low) to blue (high) in the plot.

We identified 2,533 tissue-specific genes: 569 expressed in young fruit, 775 expressed in the root, 247 expressed in the shoot, 298 expressed in the leaf, and 644 expressed in the flower (Figure 5A). KEGG enrichment analyses of these tissue-specific genes showed that gene functions correlate well with the known biological roles of each tissue. That is, the pathways photosynthesis-antenna proteins (ko00196), photosynthesis (ko00195) and porphyrin and chlorophyll metabolism (ko00860) were significantly enriched in the leaf (Rich-factors of 17.55, 7.45, and 7.41 respectively) (Figure 5B), while the starch and sucrose metabolism (ko00500, *P* = 3.2 ×10^−4^) and pentose and glucuronate interconversions (ko00040, *P* = 1.8 ×10^−8^) pathways were significantly enriched in flowers (Figure 5C). Similarly, the phenylpropanoid metabolism (ko00940, *P* = 1.62 ×10^−12^) pathway, which is required for the lignin biosynthesis (Boerjan *et al.* 2003) was the most enriched pathway in the root. In the young fruit, amino sugar and nucleotide sugar metabolism (ANM) pathway was highly enriched (Figure 5D). ANM products have many roles in living organisms, in particular, these products are important for maintaining and repairing cell walls (Lee *et al.* 2016) in starch storage organs (Zuo *et al.* 2010), which is consistent with the high starch content of the Chinese chestnut. Three genes (*EVM0005986*, *EVM0008813* and *EVM0017354*) were expressed only in the entire fruit (which included the burr).

## Discussion

As a perennial tree, the Chinese chestnut is highly heterozygous, meaning that genome assemblies for this species are typically highly fragmented, incomplete, and larger than they should be. Long read technologies can improve the resolution of highly repetitive regions (Waterston *et al.* 2002; Eid *et al.* 2009; Gnerre *et al.* 2011; Ono *et al.* 2013; Ghurye *et al.* 2017; Jiao *et al.* 2017; Staton *et al.* 2019), and Hi-C techniques can capture interactions over much larger genomic distances and produce scaffolds spanning a complete chromosome arm (Lajoie *et al.* 2015; Putnam *et al.* 2016; Jia *et al.* 2019; Liang *et al.* 2019). Here, combining the advantages of both technologies coupled with high sequencing depth, we got the final genome assembly of 688.93 Mb, which was 104.87 Mb smaller than initial SMRT assembly. Compared with previous published genomes (Staton *et al.* 2019; Xing *et al.* 2019) (Table 3), the genomic continuity has been greatly improved, ex. contig N50 length were three times longer (2.83 Mb *vs.* 944 Kb); the contig number reduced more than three times (671 *vs.* 2,707). The total scaffold number was 112, and 687.3 Mb (99.75%) sequences were anchored onto chromosomes. The previous published genome assembly lacked correction based on Hi-C data, thus no chromosome location information, and the size was 785.5 Mb, which is almost the same as our initial SMRT assembly (793.80 Mb). Collectively, we believe that the assembly in this study is the most contiguous and highest quality available for *C. mollissima* so far.

According to comparative genomic analyses of *C. mollissima* and eight other plant species (*O. sativa*, *S. tuberosum*, *A. thaliana*, *Q. robur*, *C. glauca*, *J. regia*, *P. trichocarpa*, and *P. euphratica*), the specific genes of *C. mollissima*, such as self-incompatibility, plant-pathogen interaction pathway, starch and sucrose metabolism will provide clues for understanding the adaptive evolution and nutritive value of Chinese chestnut. Phylogenetic tree represents a close relationship between *C. mollissima* and *Q. robur*, which was consistent with previous results (Staton *et al.* 2019; Xing *et al.* 2019). Due to the different species selected, the estimated divergence time would be different, and the time would be larger when *A. thaliana* and *O.sativa* were simultaneously used to estimate the divergence time. In this study, the divergence times between the distant species are similar with Zhang *et al.* (2017), and of *C. mollisima* and *Q. robur*, the time is slightly different (∼18.3 Mya *vs.* ∼13.62 Mya) with the result of Xing *et al.* (2019). The Circos plot in Figure 4 illustrates the high degree of collinearity at the whole chromosome level between *C. mollissima* and *Q. robur*, thus, the gene/trait information can potentially be shared between the two species.

We performed RNA-Seq analysis of Chinese chestnut tissues to identify genes specifically expressed in certain tissues, including the root, stem, leaf, flower (inflorescence) and fruit. Most genes were found to be highly expressed in all evaluated tissues, whereas the others only expressed in one or a few tissues, indicating they may play different roles in the regulation of related pathways (Mascher *et al.* 2017; Zhang *et al.* 2017; Jia *et al.* 2019; Liang *et al.* 2019). We identified three genes (*EVM0005986*, *EVM0008813* and *EVM0017354*) that were expressed in the complete fruit but not in the root, shoot, leaf, flower or peeled fruit. Therefore, we assumed that these genes were expressed only in the burrs. In the SwissProt database, *EVM0008813* is annotated as root meristem growth factor 3 in *Arabidopsis thaliana*. Thus, this gene may be associated with burr thickness or spine development. In future studies, we will further characterize these genes in order to better understand burr trait development in the Chinese chestnut.

## Conclusion

In this study, we used an integrated PacBio, Illumina and Hi-C sequencing approach to assemble the most contiguous and high-quality reference genome sequence for *C. mollissima* known to date. The assembly constructed herein will be useful for studies aiming at deciphering the structure and evolution of complex genomes, including *C. mollissima* and other related plant species. In addition, specific gene expression patterns in *C. mollissima* will support in-depth genetic analyses of traits of interest in this species, such as disease resistance, burr development, tolerance to adverse growing conditions, and production of highly nutritious nuts.
